# Supplementary material for: Immune Checkpoint Inhibitors in Pre-Treated Gastric Cancer Patients: Results from a Literature-Based Meta-Analysis
Source: Int J Mol Sci. 2020 Jan 10;21(2):448. doi: 10.3390/ijms21020448 (PMC7013524; doi:10.3390/ijms21020448)
Supplement: Supplementary file 1 [file ijms-21-00448-s001.pdf]

Supplementary file 1. Search strategy

PubMed:

#10 Search (((gastric cancer[Title/Abstract] OR immunotherapy[Title/Abstract] OR PD-1[Title/Abstract] OR PD-L1[Title/Abstract] OR randomized[Title/Abstract]))) AND (((((((((checkpoint) OR ICB))) OR (((((pembrolizumab) OR lambrolizumab) OR Keytruda) OR MK-3475))) OR ((((((nivolumab) OR MDX-1106) OR ONO-4538) OR BMS-936558) OR Opdivo))) OR (((((((atezolizumab) OR anti-PDL1) OR MPDL3280A) OR Tecentriq) OR RG7446) OR RG-7446))) OR ((((((ipilimumab) OR MDX-CTLA-4) OR Yervoy) OR MDX-010) OR MDX010))) OR Durvalumab) OR (((((((CTLA-4 Antigen[MeSH Terms]) OR CTLA-4) OR CTLA4) OR CD152) OR Cytotoxic T-LymphocyteAssociated Antigen 4) OR Cytotoxic T-Lymphocyte Antigen 4)))

#9 Search (((((((((checkpoint) OR ICB))) OR (((((pembrolizumab) OR lambrolizumab) OR Keytruda) OR MK-3475))) OR ((((((nivolumab) OR MDX-1106) OR ONO-4538) OR BMS-936558) OR Opdivo))) OR (((((((atezolizumab) OR anti-PDL1) OR MPDL3280A) OR Tecentriq) OR RG7446) OR RG-7446))) OR ((((((ipilimumab) OR MDX-CTLA-4) OR Yervoy) OR MDX-010) OR MDX010))) OR Durvalumab) OR (((((((CTLA-4 Antigen[MeSH Terms]) OR CTLA-4) OR CTLA4) OR CD152) OR Cytotoxic T-LymphocyteAssociated Antigen 4) OR Cytotoxic T-Lymphocyte Antigen 4))

#8 Search ((((((CTLA-4 Antigen[MeSH Terms]) OR CTLA-4) OR CTLA4) OR CD152) OR Cytotoxic T-LymphocyteAssociated Antigen 4) OR Cytotoxic T-Lymphocyte Antigen 4)

#7 Search Durvalumab

#6 Search (((((ipilimumab) OR MDX-CTLA-4) OR Yervoy) OR MDX-010) OR MDX010)

#5 Search ((((((atezolizumab) OR anti-PDL1) OR MPDL3280A) OR Tecentriq) OR RG7446) OR RG-7446)

#4 Search (((((nivolumab) OR MDX-1106) OR ONO-4538) OR BMS-936558) OR Opdivo)

#3 Search ((((pembrolizumab) OR lambrolizumab) OR Keytruda) OR MK-3475)

#2 Search ((checkpoint) OR ICB)

#1 Search (gastric cancer[Title/Abstract] OR immunotherapy[Title/Abstract] OR PD-1[Title/Abstract] OR PD-L1[Title/Abstract] OR randomized[Title/Abstract])

Embase:

#11 #1 AND #10

#10 #2 OR #3 OR #4 OR #5 OR #6 OR #7 OR #8 OR #9

#9 'cytotoxic t lymphocyte antigen 4'/exp OR 'cd152 antigen':ti,ab OR 'ctla 4':ti,ab OR 'ctla 4 antigen':ti,ab OR 'ctla-4 antigen':ti,ab OR 'antigen cd152':ti,ab OR 'ctla4':ti,ab OR 'cytotoxic t lymphocyte antigen 4':ti,ab OR 'cytotoxic t lymphocyte associated antigen 4':ti,ab

#8 'ctla-4':ab,ti OR 'ctla4':ab,ti OR 'cd152':ab,ti OR 'cytotoxic t-lymphocyte associated antigen 4':ab,ti OR 'cytotoxic t-lymphocyte antigen 4':ab,ti

#7 'durvalumab':ab,ti

#6 'ipilimumab':ab,ti OR 'mdx-ctla-4':ab,ti OR 'yervoy':ab,ti OR 'mdx-010':ab,ti OR 'mdx010':ab,ti

#5 'atezolizumab':ab,ti OR 'anti-pdl1':ab,ti OR 'mpdl3280a':ab,ti OR 'tecentriq':ab,ti OR 'rg7446':ab,ti OR 'rg-7446':ab,ti

#4 'nivolumab':ab,ti OR 'mdx-1106':ab,ti OR 'ono-4538':ab,ti OR 'bms-936558':ab,ti OR 'opdivo':ab,ti

#3 'pembrolizumab':ab,ti OR 'lambrolizumab':ab,ti OR 'keytruda':ab,ti OR 'mk-3475':ab,ti

#2 'checkpoint':ab,ti OR 'icb':ab,ti

#1 'gastric cancer':ab,ti OR 'immunotherapy':ab,ti OR 'PD-1':ab,ti OR 'PD-L1':ab,ti OR 'adverse effects':ab,ti

Web of Sciences:

TS=(checkpoint OR ICB OR pembrolizumab OR lambrolizumab OR Keytruda OR MK-3475 OR nivolumab OR MDX-1106 OR ONO-4538 OR BMS-936558 OR Opdivo OR atezolizumab OR anti-PDL1 OR MPDL3280A OR Tecentriq OR RG7446 OR RG-7446 OR ipilimumab OR MDX-CTLA-4 OR Yervoy OR MDX-010 OR MDX010 OR Durvalumab OR "CTLA-4 Antigen" OR CTLA-4 OR CTLA4 OR CD152 OR Cytotoxic "T-LymphocyteAssociated Antigen 4" OR "Cytotoxic T-Lymphocyte Antigen 4") AND TS=(gastric cancer OR immunotherapy OR PD-1 OR PD-L1 OR randomized)

Study or Subgroup	log[Hazard Ratio]	SE	Weight	Hazard Ratio IV, Random, 95% CI
ATTRACTION-2	-0.462	0.1078	32.6%	0.63 [0.51, 0.78]
JAVELIN Gastric 300	0.0953	0.1024	33.1%	1.10 [0.90, 1.34]
KEYNOTE-061	-0.0619	0.0887	34.3%	0.94 [0.79, 1.12]
**Total (95% CI)**			**100.0%**	**0.87 [0.64, 1.18]**

Heterogeneity: Tau² = 0.06; Chi² = 14.91, df = 2 (P = 0.0006); I² = 87%
Test for overall effect: Z = 0.90 (P = 0.37)

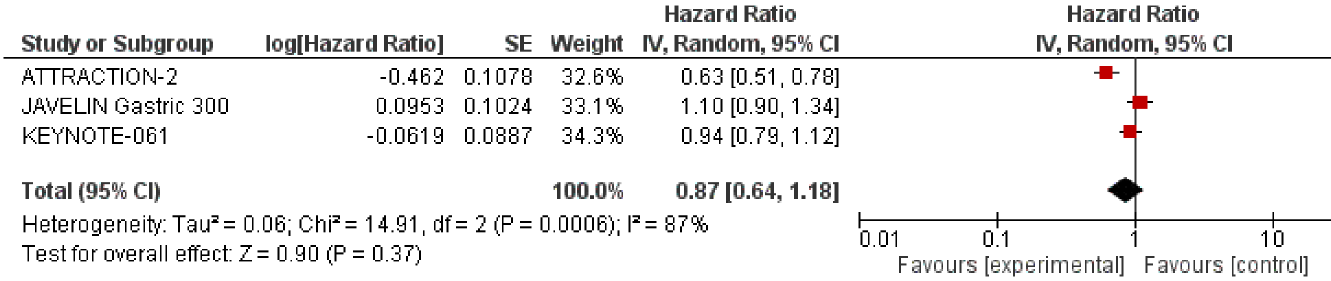

Figure S1. Forest plots of hazard ratios (HRs) for overall survival (OS). The Chi-squared test showed low heterogeneity between the trials. The random-effects model was used.

Study or Subgroup	log[Hazard Ratio]	SE	Weight	Hazard Ratio IV, Random, 95% CI
ATTRACTION-2	-0.5108	0.1033	33.3%	0.60 [0.49, 0.73]
JAVELIN Gastric 300	0.5481	0.108	33.2%	1.73 [1.40, 2.14]
KEYNOTE-061	0.3988	0.0896	33.6%	1.49 [1.25, 1.78]
**Total (95% CI)**			**100.0%**	**1.16 [0.62, 2.17]**

Heterogeneity: Tau² = 0.30; Chi² = 62.18, df = 2 (P < 0.00001); I² = 97%
Test for overall effect: Z = 0.45 (P = 0.65)

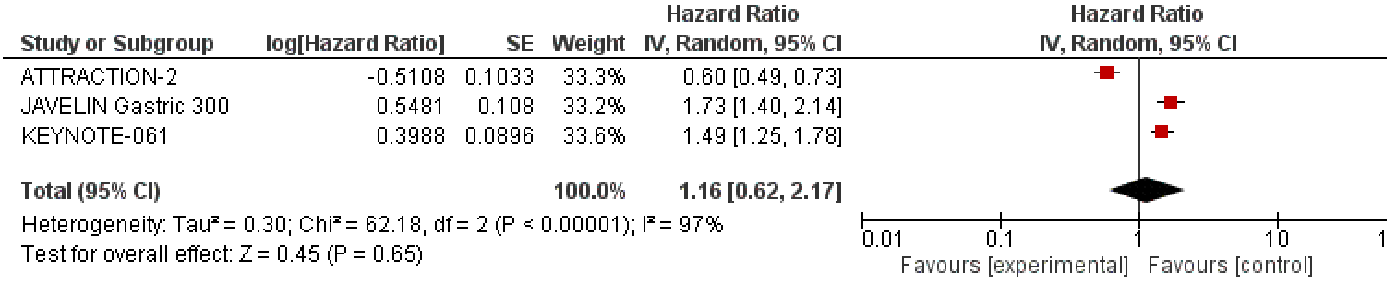

Figure S2. Forest plots of hazard ratios (HRs) for progression free survival (PFS). The Chi-squared test showed heterogeneity between the trials. The random-effects model was used.

Study or Subgroup	Experimental Events	Experimental Total	Control Events	Control Total	Weight	Risk Ratio M-H, Random, 95% CI
ATTRACTION-2	141	330	43	161	31.3%	1.60 [1.20, 2.13]
JAVELIN Gastric 300	90	184	131	177	33.9%	0.66 [0.56, 0.78]
KEYNOTE-061	155	294	232	276	34.8%	0.63 [0.56, 0.71]
**Total (95% CI)**		**808**		**614**	**100.0%**	**0.86 [0.54, 1.36]**
Total events	386		406			

Heterogeneity: Tau² = 0.16; Chi² = 39.45, df = 2 (P < 0.00001); I² = 95%
Test for overall effect: Z = 0.66 (P = 0.51)

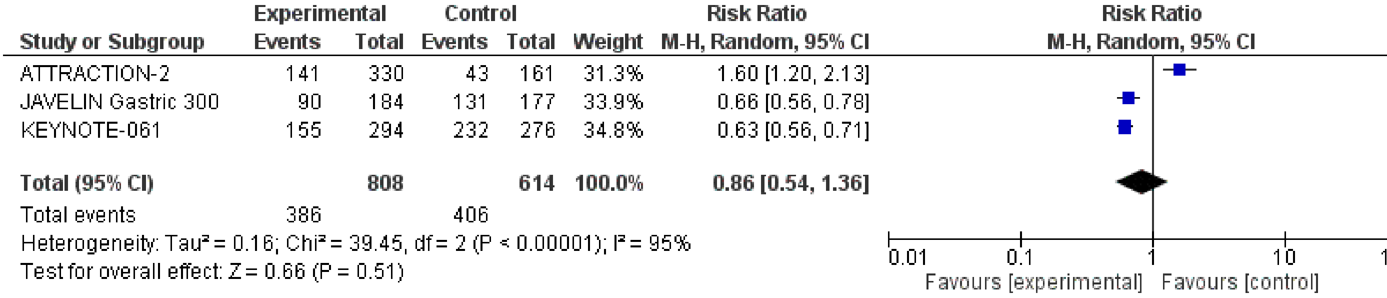

Figure S3. Forest plots of risk ratio (RR) of immune checkpoint inhibitors compared to control arm for all grade treatment-related adverse events.

Study or Subgroup	Experimental Events	Experimental Total	Control Events	Control Total	Weight	Risk Ratio M-H, Random, 95% CI
ATTRACTION-2	34	330	7	161	29.9%	2.37 [1.07, 5.23]
JAVELIN Gastric 300	18	184	69	177	34.2%	0.25 [0.16, 0.40]
KEYNOTE-061	42	294	96	276	35.8%	0.41 [0.30, 0.57]
**Total (95% CI)**		**808**		**614**	**100.0%**	**0.59 [0.22, 1.55]**
Total events	94		172			

Heterogeneity: Tau² = 0.66; Chi² = 23.03, df = 2 (P < 0.00001); I² = 91%
Test for overall effect: Z = 1.08 (P = 0.28)

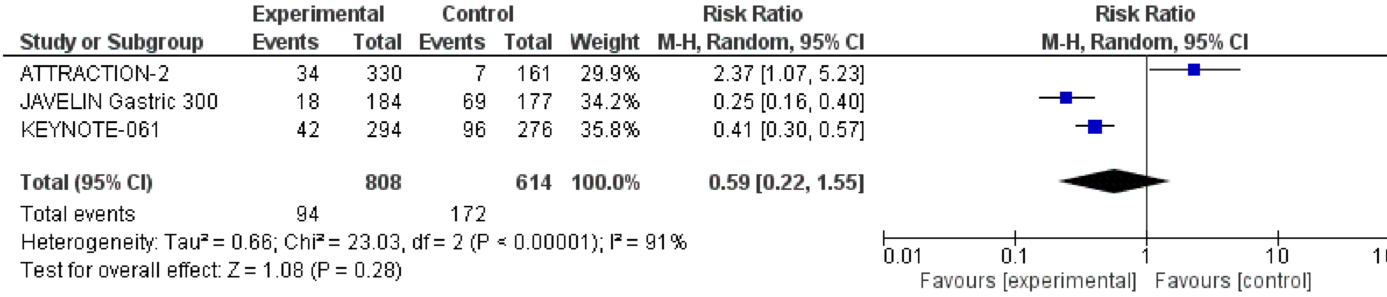

Figure S4. Forest plots of risk ratio (RR) of immune checkpoint inhibitors compared to control arm grade ≥3 treatment-related adverse events.
